# Spatial Heterogeneity in Soil Microbial Communities Impacts Their Suitability as Bioindicators for Evaluating Productivity in Agricultural Practices

**DOI:** 10.3390/microorganisms13051160

**Published:** 2025-05-20

**Authors:** Guoqiang Li, Xuanjing Li, Ting Jin, Muyilan Jiang, Peng Shi, Gehong Wei

**Affiliations:** State Key Laboratory for Crop Stress Resistance and High-Efficiency Production, Shaanxi Key Laboratory of Agricultural and Environmental Microbiology, College of Life Sciences, Northwest A&F University, Yangling 712100, China2019013669jmyl@nwafu.edu.cn (M.J.)

**Keywords:** agricultural practices, soil bacterial community, spatial heterogeneity, co-occurrence network, key indicator species

## Abstract

Soil microorganisms are increasingly recognized as critical regulators of farmland soil fertility and crop productivity. However, the impacts of spatial heterogeneity in soil microbial communities on bioindicators for evaluating agricultural practices remain poorly understood and warrant further validation. Through field experiments, this study investigated the differential effects of agricultural practice treatments on soil properties and bacterial communities between two main farmland soil compartments: intra-row and inter-row. Additionally, we explored the potential correlations between key taxa and soil properties, as well as maize biomass. Results revealed marked disparities in soil properties, bacterial community compositions, and co-occurrence network patterns between intra-row and inter-row soils. Agricultural practice treatments exerted significant impacts on bacterial community structures and network topological features in both intra-row and inter-row soils. Subsequent correlation analysis demonstrated strong relationships between soil properties and most keystone species. In addition, 42 and 41 indicator species were identified in intra-row and inter-row soils, respectively, including shared genera such as *Solirubrobacter*, *Blastococcus*, *Iamia*, *Conexibacter*, and *Lysobacter*. Notably, 22 key indicator species in intra-row soils displayed significant positive/negative correlations with maize biomass, whereas only 4 key indicator species showed negative correlations in inter-row soils. These findings highlight differential responses of bacterial communities to agricultural practices in distinct soil compartments. The intra-row soils harbored more bacterial taxa significantly associated with maize biomass, while the inter-row soils better reflected the effects of agricultural interventions. This study confirms the spatial variability of microbial communities as effective bioindicators for evaluating agricultural practice strategies. Identification of compartment-specific indicators provides novel microbiological insights into supporting precision agriculture practices.

## 1. Introduction

Soil organisms, particularly soil microorganisms, are critical components of agroecosystems [1,2,3]. As core regulators of soil functions, these microorganisms promote organic matter decomposition [4,5], regulate biogeochemical cycles and nutrient transformation [6,7], and modulate plant immunity [8,9,10]. Through multiple ecological processes, they play irreplaceable roles in maintaining soil fertility, promoting plant growth, and sustaining the stability of agroecosystems in farmlands [11,12,13]. Given the crucial role of soil microorganisms, many studies have regarded microbial characteristics as important bioindicators for evaluating the impacts of long-term or short-term agricultural practices on soil health and productivity [14,15]. For example, microbial biomass carbon and nitrogen are widely employed to assess soil nutrient cycling capacity [16], while microbial community diversity indices are routinely used to estimate ecosystem stability and resilience in response to agricultural disturbances [17,18]. Compared to soil physicochemical properties, soil microbial communities are more sensitive and respond more rapidly to environmental changes [19,20,21], providing advantages in the dynamic assessment of short-term soil productivity [15,22,23]. In particular, bacterial communities, characterized by rapid reproduction rates, high metabolic activity, and sensitivity to environmental disturbances [24,25], serve as effective bioindicators for reflecting dynamic changes in soil health and productivity [11,12,26]. Through the detection of key bioindicators, the dynamics of soil productivity can be quickly determined during crop growth [26,27]. Tillage and management measures can be adjusted in a timely manner based on the soil nutrient supply status, ensuring that the nutrient provision synchronizes with crop demands at different growth stages, which enhances nutrient use efficiency, promotes crop growth, and increases crop yield [1,28,29].

The structure, composition, and functions of soil microbial communities are influenced by various factors, such as nutrient distribution and water availability, exhibiting spatial heterogeneity [30,31,32,33]. In farmland ecosystems, accumulating studies highlight substantial spatial heterogeneity of microbial communities across distinct ecological compartments (e.g., rhizosphere and bulk soil), which are closely associated with plant root activity and agricultural strategies [34,35]. Root exudates provide carbon sources and energy, shaping the specific rhizosphere environments created by plant hosts [36,37]. Microbial assemblages colonizing different root zones of plants, forming structural and functional disparities in microbial communities, play indispensable roles in plant health and ecological functions [38,39], including host nutrient acquisition [40], stress tolerance enhancement [37,41,42], and carbon/nitrogen cycling regulation [6,36,43]. In bulk soil, the spatial heterogeneity of microbial communities is related to soil properties [44,45,46,47], and the stability of their community structure and ecological functions are substantially influenced by agricultural practices [43,48]. Studies have indicated that agricultural practices, such as balanced application of inorganic/organic fertilizers [2,49] and the promotion of crop diversification (e.g., legume-based rotations, intercropping systems) [46,47,50,51,52,53], influence the original balance of soil microbial communities and reshape microbial habitats [29,49,51,54,55], thereby augmenting soil nutrient and water availability. Agricultural interventions can modify interspecies interactions, effectively enhancing soil nutrient cycling and enriching functional microbial guilds (e.g., nitrogen-fixing, phosphate-solubilizing, and other functional microorganisms) [42,56]. However, current research still lacks a systematic understanding of the mechanisms driving spatial heterogeneity in microbial communities across different practices, as well as the impacts of these mechanisms on crop growth. Further exploration of these processes could provide insights into precision agriculture strategies aimed at optimizing soil fertility and crop performance.

Agricultural practices affect the distribution and function of microbial communities by altering soil physicochemical properties and biological activities, playing an important regulatory role in soil nutrients and crop productivity [57,58]. With the advancement of precision agriculture, agricultural practice strategies have been increasingly implemented to preserve or enhance microbial diversity, contributing to functional diversification by improving soil quality [53], alleviating nutrient imbalances [44,59], and mitigating soil health degradation [60]. Spatial heterogeneity determines the differential responses of microbial communities in distinct soil compartments to agricultural practices [34,35], bringing challenges when applying bioindicators, especially in evaluating soil productivity. At the farmland scale, the relationship in different spatial compartments between microbial communities and crop growth, affected by root exudates and agricultural measures to varying degrees, exhibits diversity [17,18,27,35,36,37]. Therefore, in practical evaluation processes, the representativeness of samples becomes one of the key factors determining the accuracy of assessments. If sampling fails to adequately cover diverse environments, such as by selecting only a single sampling point or mixing samples in a simple manner, it may obscure the spatial heterogeneity of microorganisms, hindering the characterization of the true association between microbial communities and soil productivity. However, most current studies on microbial indicators for soil health and productivity have not accounted for soil heterogeneity in farmlands [53,61,62]. We argue that the reliability of using microbial indicators to assess soil health and productivity requires thorough consideration of spatial heterogeneity, clarification of the relationships between microbial characteristics in different spatial compartments and crop growth stages, and the acquisition of representative samples. This approach enables the screening of more reliable microbial indicators, providing a robust basis for scientifically assessing soil productivity and guiding agricultural practices.

Although the importance of microbial indicators in soil health and productivity assessments has been widely recognized [63,64], the microecological characteristics of multiple soil compartments and the spatial heterogeneity in microbial responses to various agricultural practices remain to be further explored. Understanding which microbial functional groups are more strongly associated with crop growth and more suitable as core bioindicators for evaluating soil productivity is essential for efficiently utilizing soil microbial resources. Therefore, a field experiment was conducted in a rainfed agricultural region of northern China, with intra-row and inter-row soils as the spatial compartment representatives, involving four treatments: maize monoculture without fertilization (Con_Mono), maize/soybean intercropping without fertilization (Con_Inter), maize monoculture with fertilization (Fer_Mono), and maize/soybean intercropping with fertilization (Fer_Inter). We hypothesized that soil bacterial communities exhibit significant spatial disparities between intra-row and inter-row soils in response to agricultural practices, with inter-row soils being more strongly influenced by agricultural practices and the effects are mitigated in intra-row soils. Bacterial taxa significantly associated with crop growth also differ notably between these two soil compartments, exhibiting distinct applicability characteristics when assessing soil productivity. Using high-throughput sequencing, we aimed to characterize spatial differences in bacterial community patterns between intra-row and inter-row soils, compare the differences in maize growth, soil properties, and bacterial communities under four agricultural practices, and analyze potential correlations between key taxa and soil/maize parameters. We aimed to investigate the differences and the potential influencing factors in the spatial heterogeneity of the microbial community structure between intra-row and inter-row soils and explore which farmland soil compartment is more suitable for productivity assessments.

## 2. Materials and Methods

### 2.1. Site Description and Experimental Design

Field experiments were conducted in the vicinity of Changwu Agro-Ecological Experimental Station in Shaanxi Province, China (35°14′47″ N, 107°41′45″ E). The experimental site is located in the hilly-gully region of the southern Loess Plateau, characterized by a semi-arid continental monsoon climate. Reliant on natural precipitation, agricultural systems are dominated by annual monocropping of maize (*Zea mays* L.) or wheat (*Triticum aestivum* L.). The soil, developed from loess deposits, is classified as silty loam (Cumulic Haplustoll according to USDA Soil Taxonomy) with medium fertility.

**Figure 1:**
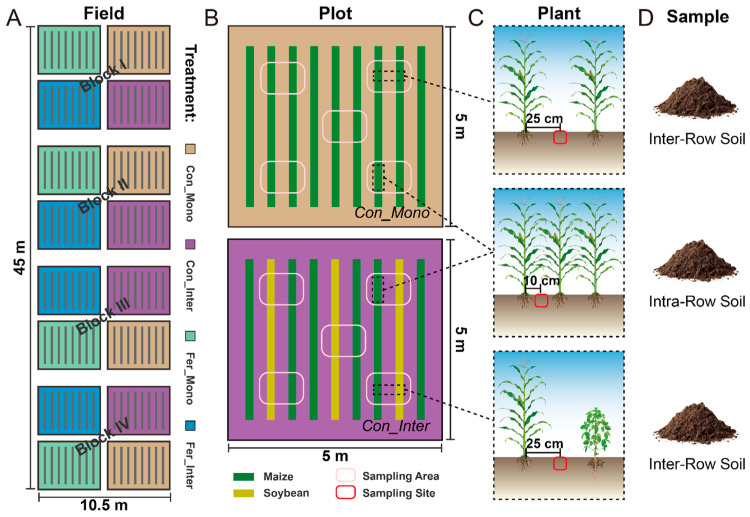
Schematic diagram of the experimental design, field layout, and sample collection. (**A**) Layout of field experiments. Each individual plot is outlined in black lines. These plots, representing four treatments (yellow: Con_Mono, purple: Con_Inter, green: Fer_Mono, blue: Fer_Inter), were arranged semi-randomly in four blocks. (**B**) Schematics of Con_Mono (**top**) and Con_Inter (**bottom**). Green and yellow bands represent maize and soybean rows, respectively. Pink ovals outline approximate sampling areas in each plot. (**C**,**D**) Sampling sites and types for soil collection. Red ovals indicate sampling sites for intra-row and inter-row soils.

Field experiments started in late April 2017, employing a split-plot design with four replications. Treatments were established by combining fertilization (non-fertilized vs. fertilized) and cropping practices (monoculture vs. intercropping), resulting in four treatment combinations: maize monoculture without fertilization (Con_Mono), maize/soybean intercropping without fertilization (Con_Inter), maize monoculture with fertilization (Fer_Mono), and maize/soybean intercropping with fertilization (Fer_Inter). Plots representing these four treatments were arranged in a semi-random manner, with each plot separated by soil ridges. Chemical fertilizers containing nitrogen, phosphate, and potassium were applied to the fertilized treatments. The maize/soybean intercropping followed a cropping practice with soybean rows planted between every two maize rows. In Con_Mono and Fer_Mono plots, nine maize rows were planted at inter-row spacings of 50 cm and intra-row spacings of 25 cm, achieving a density of 170 plants per plot. For Con_Inter and Fer_Inter, their plots featured six maize and three soybean rows. Each soybean row was planted with an intra-row spacing of 13 cm, resulting in densities of 99 soybean plants and 114 maize plants per plot. A detailed schematic of the experimental design and field layout is presented in Figure 1.

### 2.2. Fertilization and Management

Field experiments were conducted over two consecutive years (2017–2018). Annual fertilization was applied according to treatments. Chemical fertilizers were applied pre-planting for both maize and soybean in the fertilized plots (Fer_Mono and Fer_Inter). The fertilizer sources included urea (46% N), calcium superphosphate (16% P_2_O_5_), and potassium sulfate (51% K_2_O), applied at annual rates of 180 kg N, 120 kg P_2_O_5_, and 100 kg K_2_O ha^−1^, respectively. All fertilizers were manually spread over the soil surface and uniformly plowed into the cultivated layer using a rotary tiller. Maize (Zhengdan 958) and intercropped soybean (Zhonghuang 13) were sown in the first week of May using a hand-powered hole-seeding machine. Crop growth relied solely on natural precipitation with no supplementary irrigation. Weed control measures were manually performed three to four times annually. All other agronomic practices were standardized and consistently applied across all experimental plots.

### 2.3. Sample Collection and Processing

Plant and soil samples were collected to determine maize growth, soil properties, and microbial community. To minimize variability, we standardized sampling by designating specific maize rows within plots sharing identical sampling areas and spatial configurations as experimental rows. Soybean rows in Con_Inter/Fer_Inter plots and the corresponding maize rows in Con_Mono/Fer_Mono plots were defined as supplementary rows. All sampling was strictly confined to these predefined sampling areas and rows, which were located more than 1 m away from the edges of each plot to minimize potential edge effects during sampling. This standardized arrangement effectively ensured consistent plant and soil sampling across different treatments.

Plant and soil samples were obtained simultaneously in the late vegetative stages of 2017 and 2018, which shared the same growth period in both years. Three maize plants were randomly selected from each experimental plot and dug up using a shovel. A total of 96 plant samples (4 treatments × 3 sample replicates × 4 block replicates × 2 years) were obtained. Plant height was measured with a ruler. The aboveground parts of these plants were separated from the root systems and oven-dried at 85 °C to constant weight for biomass determination.

Soil samples were collected from sampling areas demarcated in each plot to obtain soils from two compartments. Soils outside maize rows (inter-row soil) were collected from the intermediate zone between two adjacent plant rows, while soils inside maize rows (intra-row soil) were collected from the maize root zone between two plants in experimental rows (Figure 1C,D). Five soil cores (5 cm diameter) were randomly collected using a soil drill and combined into one composite soil sample per plot, resulting in 64 soil samples (4 treatments × 2 soil compartments × 4 block replicates × 2 years). Soil samples were stored in individual polyethylene bags with ice packs and transported to the laboratory for subsequent processing. All samples were divided into two subsamples: one portion was stored at −80 °C for DNA extraction, and the other was stored at −20 °C for soil physicochemical analysis. Standard soil testing procedures and previous reports were followed to quantify: soil pH (1:2.5 soil:water suspension), total carbon/nitrogen (TC/TN, Elementar vario MAX cube CNS analyzer, Elementar, Langenselbold, Germany), dissolved organic carbon (DOC, 0.5 mol L^−1^ K_2_SO_4_ extraction-Elementar vario TOC cube TOC analyzer, Elementar, Langenselbold, Germany), nitrate-/ammonium-nitrogen (NO_3_^−^-N/NH_4_^+^-N, 2 mol L^−1^ KCl extraction–colorimetric quantification), and microbial biomass carbon/nitrogen (MBC/MBN, chloroform fumigation–extraction method).

### 2.4. DNA Extraction, PCR, and High-Throughput Sequencing

Total genomic DNA was extracted from soil samples using the FastDNA^®^ SPIN Kit for Soil (MP Biochemicals, Solon, OH, USA) following the manufacturer’s protocols. DNA concentration and purity were assessed using Nanodrop 2000 (Thermo Scientific, Waltham, MA, USA) and 1% (*w*/*v*) agarose gel electrophoresis (Bio-Rad, Hercules, CA, USA). The V3–V4 region of the bacterial 16S rRNA gene was amplified using the primer pairs 338F (5′-ACTCCTACGGGAGGCAGCAG-3′) and 806R (5′-GGACTACHVGGGTWTCTAAT-3′) [65]. PCR products were purified and pooled into libraries at equimolar concentrations. The libraries were then sequenced on an Illumina MiSeq PE300 platform (Illumina Inc., San Diego, CA, USA). PCR amplification, library preparation, and high-throughput sequencing were performed according to the standard protocols provided by a biotechnology company (Novogene, Shanghai, China).

### 2.5. Bioinformatics Analysis

Raw sequence datasets were mainly processed and analyzed using the DADA2 bioinformatics pipeline [66]. Based on the DADA2 workflows, low-quality sequences were truncated and the sequence length was trimmed, respectively. Forward and reverse sequences with more than three expected errors were discarded. After sequence denoising, pair merging, and chimera detection, amplicon sequence variants (ASVs) were determined from raw sequence datasets. The taxonomy of these bacterial ASVs was assigned using the RDP Classifier (version 2.11) [67] with an 80% confidence threshold based on the Silva database (version 128, https://www.arb-silva.de (accessed on 14 May 2023)) [68]. Representative sequences were aligned using MUSCLE (version 5.1) [69] and phylogenetic trees were generated from the aligned sequences using FastTree (version 2.1) [70]. Based on taxonomic assignments, the ASVs not assigned to the kingdom Bacteria and those assigned to Archaea, mitochondria, and chloroplasts were removed. To minimize sequencing errors and low-prevalence features for downstream analysis, those bacterial ASVs of less than two counts were discarded. The resulting count table was normalized to the sample with the lowest number of sequences, and subsequent analyses were based on this ASV count table.

### 2.6. Statistical Analysis

All statistical analyses were performed in the R environment (version 3.6.3; R Core Team 2020). Significant differences among treatments were identified at a significance level of *p* < 0.05 unless otherwise indicated. Non-parametric tests were performed to analyze the significance of differences between variables (compartment and treatment) in maize biomass, soil properties, dominant phyla, and alpha diversity.

Based on normalized ASV count table, alpha diversity was characterized by ASV richness, the Shannon–Wiener index, and phylogenetic diversity (PD) [71]. Beta diversity analyses of bacterial communities were calculated using the Bray–Curtis distance. Permutation multivariate analysis of variance (PERMANOVA) and analysis of similarities (ANOSIM) were used to assess the effects of variables with 10^4^ permutations in bacterial community dissimilarities [72]. Principal coordinate analysis (PCoA) was conducted to quantify major variance components [73] and constrained analysis of principal coordinates (CAP) was also used to confirm the treatment components [72]. To identify the ASVs’ responses to treatments in each compartment, we carried out indicator species analyses to calculate the point-biserial correlation coefficient of the positive association between an ASV and one or a combination of treatments. This analysis was conducted with 10^4^ permutations and was considered to be statistically significant when *p* < 0.05. Bipartite networks were constructed using the Fruchterman–Reingold layout and were used to visualize the significant ASVs obtained from the indicator species analyses [74].

Spearman correlation analyses were performed on the bacterial ASVs prevalent in more than 50% of samples, and co-occurrence networks were constructed using these ASVs with significant correlations (|*ρ*| > 0.6, FDR-adjusted *p* < 0.001). The networks were visualized using Gephi (Version 0.9.6) [74] and Cytoscape (Version 3.6.1) [75]. Topology parameters of these networks were also calculated, and the significance of these differences between compartments was assessed separately using Kolmogorov–Smirnov tests under different treatments. The network robustness was measured by natural connectivity as a function with the number of deleted nodes. In addition, the intra-module connectivity (*Zi*) and inter-module connectivity (*Pi*) of the nodes were divided into peripherals (*Zi* ≤ 2.5, *Pi* ≤ 0.62), connectors (*Zi* ≤ 2.5, *Pi* > 0.62), module hubs (*Zi* > 2.5, *Pi* ≤ 0.62), and network hubs (*Zi* > 2.5, *Pi* > 0.62) according to the values of *Zi* (2.5) and *Pi* (0.62). Nodes belonging to connectors, module hubs, and network hubs were defined as keystone species. Correlations between keystone species and soil properties, along with maize biomasses, were calculated using Spearman’s rank correlation coefficient.

## 3. Results

### 3.1. Maize Biomass and Soil Properties Across Treatments

Our results demonstrated that treatments had a significant effect on maize height and aboveground biomass across all samples. Statistical analysis revealed that both maize height and aboveground biomass were significantly greater under the Fer_Inter treatment than the Con_Mono, Con_Inter, and Fer_Mono treatments. Specifically, maize height and aboveground biomass were the lowest in Con_Mono plots (136.15 cm; 99.50 g plant^−1^), whereas they were the highest in Fer_Inter plots (235.95 cm; 208.95 g plant^−1^; Table 1).

**Figure 2:**
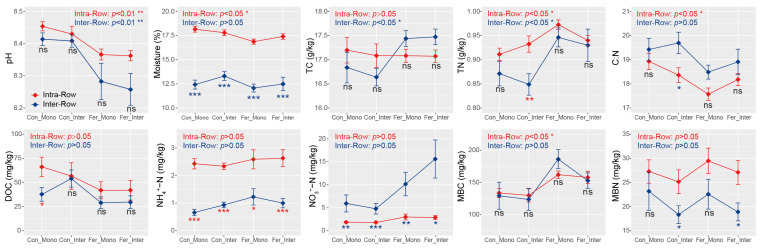
Soil properties between different treatments in intra-row and inter-row soils. Statistical significance of these differences was assessed using the Kruskal–Wallis test, and the results were presented at the top of each plot. The separate significance of differences between intra-row and inter-row soils under each treatment was assessed using the Wilcoxon rank sum test. ***: *p* < 0.001; **: *p* < 0.01; *: *p* < 0.05; ns: non-significant; TC: total carbon; TN: total nitrogen; C:N: total carbon/nitrogen ratio; DOC: dissolved organic carbon; NH_4_^+^-N: ammonium nitrogen; NO_3_^−^-N: nitrate nitrogen; MBC: microbial biomass carbon; MBN: microbial biomass nitrogen.

Significant differences in moisture, TN, C:N, NH_4_^+^-N, NO_3_^−^-N, and MBN were detected between intra-row and inter-row soils using Wilcoxon rank sum tests. Specifically, intra-row soils exhibited significantly higher moisture, TN, NH_4_^+^-N, and MBN, but lower C:N and NO_3_^−^-N compared to inter-row soils (Figure S1). Moreover, intra-row soils exhibited significant differences in pH, moisture, TN, C:N, and MBC, while inter-row soils exhibited significant differences in pH, TC, and TN among treatments. Additionally, substantial differences in moisture, NH_4_^+^-N, and NO_3_^−^-N were detected between intra-row and inter-row soils under each treatment. MBN also differed significantly between compartment soils under Con_Inter and Fer_Inter treatments (Figure 2).

### 3.2. Composition and Alpha Diversity of Soil Bacterial Communities Across Treatments

**Figure 3:**
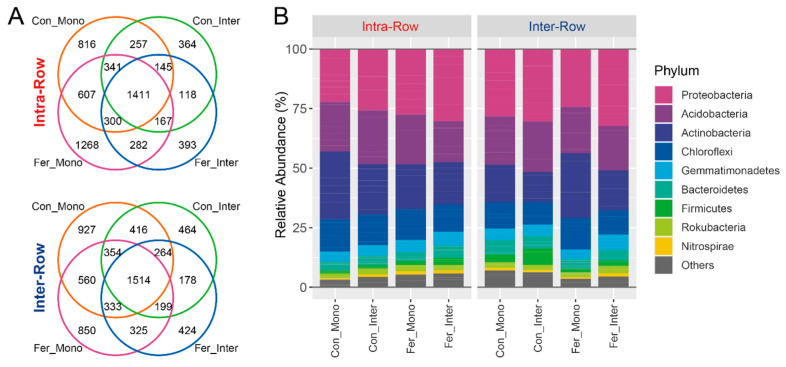
Distributions of the ASVs (**A**) and dominant phyla (**B**) of soil bacterial communities for each treatment in intra-row and inter-row soils. Phyla with relative abundances less than 1% were summarized as “Others”.

Bacterial community profiling was conducted to investigate the soil microbial communities, and amplicon sequencing was performed on soil samples collected from field experiments. After quality control, a count table with 8267 bacterial ASVs was obtained from 64 samples. The ASV count table was normalized with the minimum sequence count of the sample (11,265) to standardize sampling effort. Subsequent analysis was performed based on the normalized table. Overall, 6990 bacterial ASVs were obtained from intra-row soils while 7425 were obtained from inter-row soils. Treatments shared 1411 and 1514 bacterial ASVs in intra-row and inter-row soils, respectively (Figure 3A). These obtained ASVs belonged to 38 phyla, 95 classes, 172 orders, 232 families, and 399 genera. Most ASVs (8085 ASVs, 97.38%) could be classified at the phylum level. Proteobacteria was the most abundant phylum, comprising 27.64% of all bacterial ASVs, followed by Acidobacteria, Actinobacteria, and Chloroflexi, accounting for 20.11%, 19.87%, and 11.80%, respectively (Figure 3B).

**Figure 4:**
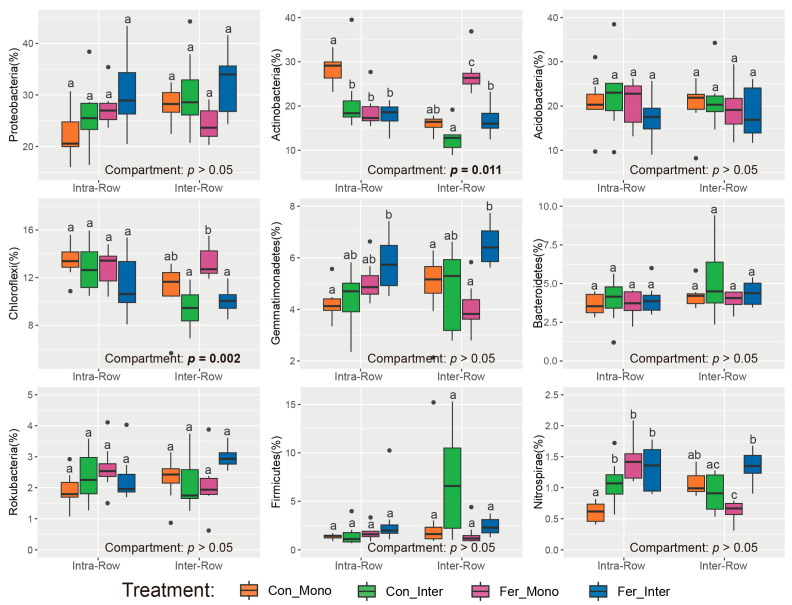
Relative abundances of dominant bacterial phyla in different treatments. Significance of differences between intra-row and inter-row soils was assessed using the Wilcoxon rank sum test. Results are presented at the bottom of each plot, with significant effects highlighted in bold. The separate significance of differences among treatments in intra-row and inter-row soils was assessed using the Kruskal–Wallis test. Different letters indicate significant differences among treatments.

Dominant phyla (>1% relative abundance) were analyzed to evaluate the effects of compartment and treatment on soil bacterial community compositions. Results showed that compartment had significant impacts on Actinobacteria and Chloroflexi, with their relative abundances in intra-row soils being significantly higher than those in inter-row soils (Figure 4). No significant differences were detected in Proteobacteria, Acidobacteria, Gemmatimonadetes, Bacteroidetes, Rokubacteria, Firmicutes, and Nitrospirae between compartment soils. Additionally, Actinobacteria, Gemmatimonadetes, and Nitrospirae significantly differed among treatments in intra-row soils. In inter-row soils, Actinobacteria, Chloroflexi, Gemmatimonadetes, Firmicutes, and Nitrospirae were significantly different among treatments (Figure 4).

**Figure 5:**
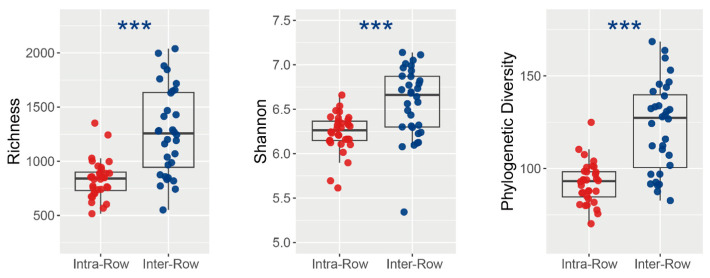
Alpha diversity of soil bacterial communities between intra-row and inter-row soils. Significance of the differences was assessed using the Wilcoxon rank sum test. ***: *p* < 0.001.

ASV richness, Shannon–Wiener index, and phylogenetic diversity (PD) were calculated to assess how bacterial alpha diversity was influenced by variables. Richness, Shannon index, and PD varied significantly between the compartment soils. Specifically, in inter-row soils, richness, Shannon index, and PD were significantly higher than those in the intra-row soils (Figure 5). Furthermore, no significant variations in richness, Shannon index, and PD were observed among treatments in intra-row soils. Treatments slightly differed the richness, whereas significantly differed the Shannon index and PD in inter-row soils (Table 2).

### 3.3. Bacterial Community Structure in Soils

**Figure 6:**
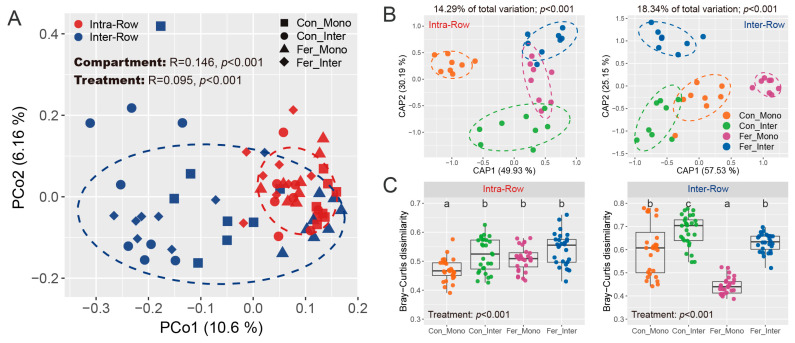
General patterns of bacterial communities across soil samples. (**A**) PCoA shows bacterial community structures based on Bray–Curtis distance. Similarity of compartment and treatment was estimated via ANOSIM and presented at the top of the plot. Colored circles represent different compartment soils, while shapes represent the treatments, with 80% confidence ellipses shown around each compartment. (**B**) CAP shows the treatment patterns in intra-row and inter-row soils, respectively. The CAP analyses were constrained by “treatment”. The explained fraction of total variance is indicated above the plots. The percentage of variation shown on each axis refers to the explained fraction of total variance. Colored circles represent the four treatments, with 80% confidence ellipses shown around each treatment. (**C**) Bray–Curtis dissimilarity of bacterial communities among different treatments in intra-row and inter-row soils. The significance of differences among treatments was evaluated using the Kruskal–Wallis test. Different letters denote significant differences among treatments.

General patterns of bacterial communities were evaluated based on Bray–Curtis distance, and the similarities of compartment and treatment were examined via permutational multivariate analysis of variance (PERMANOVA) and analysis of similarities (ANOSIM). Results showed that compartment variability altered bacterial community structures across all samples. Significant differences were observed between intra-row and inter-row soils (PERMANOVA: R^2^ = 0.052, *p* < 0.001; ANOSIM: R = 0.146, *p* < 0.001; Table S1). These differences were visualized and confirmed using principal coordinate analysis (PCoA), which showed the samples from intra-row and inter-row soils forming distinct clusters in the spatial ordination, accounting for 10.6% (PCo1) and 6.16% (PCo2) of the total variation for the first and second principal coordinate axes, respectively (Figure 6A). Constrained principal coordinate analysis (CAP) based on Bray–Curtis distance further verified these results (Figure S2A). Moreover, Bray–Curtis dissimilarity was significantly higher in inter-row soils compared to intra-row soils (Figure S2B). Furthermore, treatment effects on bacterial communities were also significant as determined by PERMANOVA and ANOSIM (PERMANOVA: R^2^ = 0.081, *p* < 0.001; ANOSIM: R = 0.095, *p* < 0.001; Table S1).

Considering the significant differences in bacterial community structure between intra-row and inter-row soils, we further examined the treatment effects in these two compartment soils to investigate the potential differences in microbial communities. PERMANOVA and ANOSIM revealed that bacterial communities presented significant differences among treatments in intra-row and inter-row soils, respectively (Intra-Row: PERMANOVA, R^2^ = 0.143, *p* < 0.001; ANOSIM, R = 0.203, *p* < 0.001; Inter-Row: PERMANOVA, R^2^ = 0.183, *p* < 0.001; ANOSIM, R = 0.221, *p* < 0.001; Table S2). Furthermore, CAP analysis was performed to visualize the treatment effects. CAP analysis showed that the Con_Mono samples were distinctly separated from the other three treatments in the spatial ordination, forming a different cluster in intra-row soils (Figure 6B). Meanwhile, Con_Inter, Fer_Mono, and Fer_Inter samples exhibited significant differences in bacterial communities from each other. In inter-row soils, treatment samples formed distinct and clear clusters in spatial ordination (Figure 6B). The total variation explained by treatment patterns was 14.29% in intra-row soils and 18.34% in inter-row soils (Figure 6B). Moreover, Bray–Curtis dissimilarities differed significantly among treatments. In intra-row soils, Con_Mono had significantly lower dissimilarity than the other three treatments (Figure 6C). In inter-row soils, all four treatments showed significant differences in Bray–Curtis dissimilarity (Figure 6C). These results confirmed that treatment had a greater impact on bacterial communities in inter-row soils.

### 3.4. Co-Occurrence Patterns of Soil Bacterial Communities Across Treatments

To investigate the co-occurrence patterns of bacterial communities in two compartment soils, co-occurrence networks of intra-row and inter-row samples were constructed. The networks consisted of 338 nodes and 948 edges in intra-row soils, compared to 411 nodes and 2478 edges in inter-row soils (Figure S3). Topological features revealed that the inter-row network exhibited a higher average clustering coefficient, containing significantly more positive correlations (Intra-Row: 924; Inter-Row: 2456), but lower average degree (Figure S3). Moreover, the intra-row network was divided into more modules, with a greater modularity than the inter-row network (Figure S3). Proteobacteria, Acidobacteria, Actinobacteria, Gemmatimonadetes, and Chloroflexi were the five most abundant phyla, accounting for 87.57% and 88.81% of total nodes in intra-row and inter-row networks, respectively (Figure S4). Based on the clustering characteristics of network nodes, the top six modules with the highest abundances were selected. In the intra-row network, Proteobacteria dominated the nodes in modules I, IV, and VI, while Actinobacteria dominated the nodes in modules II, III, and V. In the inter-row network, Proteobacteria were dominant in modules I, IV, and V, Gemmatimonadetes were dominant in module II, Chloroflexi were dominant in module III, and Actinobacteria were dominant in module VI (Figure S4).

**Figure 7:**
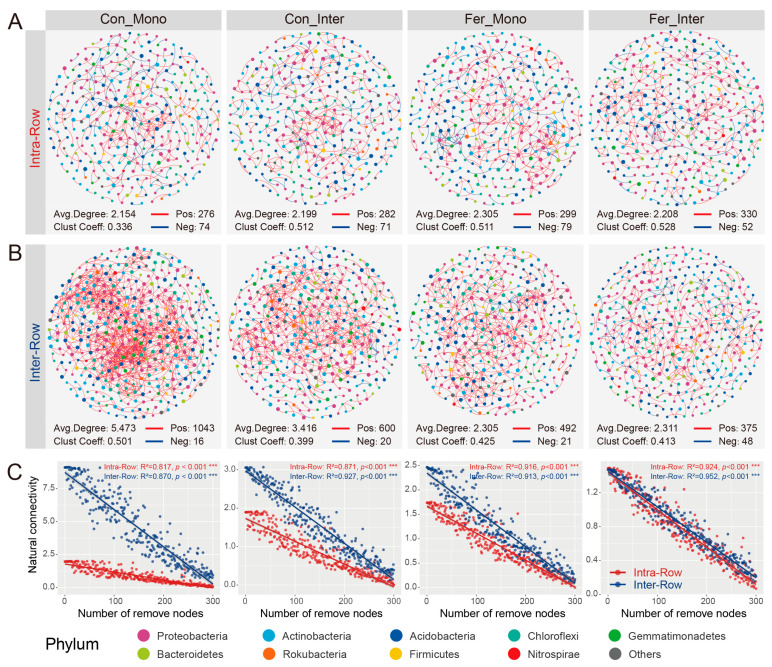
Co-occurrence patterns of bacterial communities among different treatments in intra-row and inter-row soils. (**A**,**B**) Co-occurrence networks for each treatment in intra-row (**A**) and inter-row (**B**) soils. Networks were constructed by calculating correlations among ASVs (|*ρ*| > 0.6, *p* < 0.001). Nodes are colored according to taxonomic classification at the phylum level. Colored edges represent positive (red) and negative (blue) correlations, respectively. Node sizes represent the degree of connections. Topological parameters (number of positive/negative edges, average degree, and clustering coefficient) are presented at the bottom of each plot. (**C**) Network robustness between intra-row and inter-row soils for each treatment. Results of linear models are displayed at the top of each plot. Colored circles represent intra-row (red) and inter-row soils (blue) soils, ***: *p* < 0.001.

To further compare the co-occurrence patterns among treatments, ASVs prevalent in more than 50% of the samples were selected to construct bacterial co-occurrence networks of different treatments in both intra-row and inter-row soils. In intra-row soils, the networks of the four treatments (Con_Mono, Con_Inter, Fer_Mono, and Fer_Inter) contained 325, 321, 328, and 346 nodes and 350, 353, 378, and 382 edges, respectively (Figure 7A). In inter-row soils, the networks of the four treatments included 387, 363, 343, and 366 nodes and 1059, 620, 513, and 423 edges, respectively (Figure 7B). Substantial differences were observed in the number of edges, as well as positive connections, among treatments in inter-row networks, whereas the number of positive connections among treatments showed marginal changes in intra-row networks. The negative connections in the inter-row networks of each treatment were lower compared to those in the intra-row networks (Figure 7A,B). In intra-row networks, treatments had significant effects on closeness and eigenvector centrality. The closeness and eigenvector of the Con_Inter and Fer_Inter treatments were higher than those of the Con_Mono and Fer_Mono treatments, respectively (Figure S5). In the inter-row networks, treatments significantly affected the degree, betweenness, closeness, and eigenvector. The degree, betweenness, and eigenvector were highest in the Con_Mono treatment, whereas the closeness was highest in the Fer_Inter treatment (Figure S5). Moreover, significant differences in degree, betweenness, closeness, and eigenvector between intra-row and inter-row networks were also detected for each treatment (Table S3).

Additionally, robustness reflected the stability of the co-occurrence networks. The results showed that the soil bacterial networks were relatively stable, and no significant variations were observed in the stability of the intra-row networks (Figure 7C). The robustness of the Con_Mono, Con_Inter, Fer_Mono, and Fer_Inter networks was higher than that of the intra-row networks, indicating that treatments had significant impacts on the stability of networks in the inter-row soils (Figure 7C).

### 3.5. Key Indicator Species and Their Relationships with Soil Properties and Maize Biomass Across Treatments

**Figure 8:**
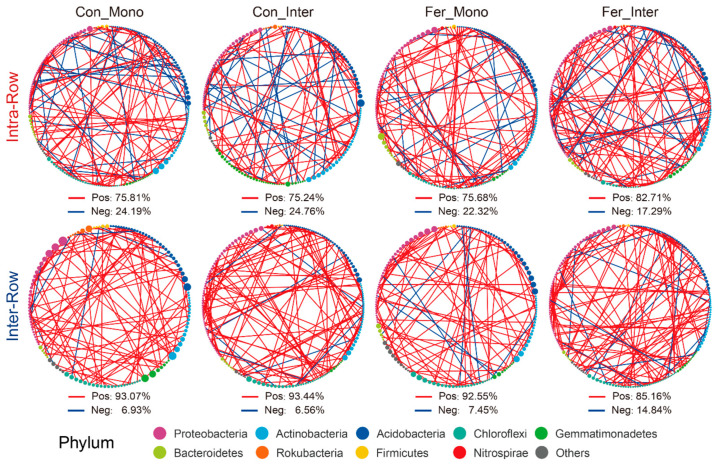
Correlation networks of keystone species for different treatments in intra-row and inter-row soils. Keystone species with correlation coefficients greater than 0.9 were screened. Nodes are colored according to taxonomic classification at the phylum level. Node sizes represent the abundance of keystone species. Colored edges represent positive (red) and negative (blue) correlations.

In treatment networks of the intra-row and inter-row soils, keystone species (critical nodes), which play pivotal roles, were identified based on the *Zi* and *Pi* values of network nodes. In intra-row networks, 200 keystone species were obtained (Figure S6A), while 211 keystone species were obtained in the inter-row networks (Figure S6B), respectively. Keystone species of each treatment with correlation coefficients greater than 0.9 were screened to construct interrelationship subnetworks. Compared to the inter-row subnetworks, keystone species in each intra-row subnetwork showed higher proportions of negative correlation connections, accounting for 24.19%, 24.79%, 22.32%, and 17.29% of the connections, respectively (Figure 8). The proportion of negative correlation connections of Fer_Inter was the highest in intra-row subnetworks but the lowest in inter-row subnetworks. These negative correlation connections mainly occurred between Proteobacteria, Actinobacteria, and Acidobacteria.

**Figure 9:**
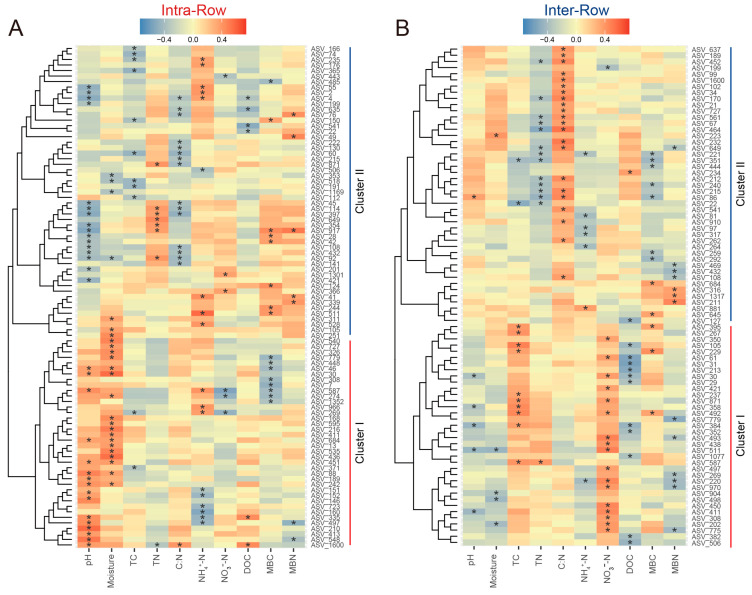
Correlations between keystone species abundance and soil properties in intra-row (**A**) and inter-row (**B**) networks. The heatmaps present only the keystone species significantly related to soil properties. Positive (red) and negative (blue) correlations are indicated by color intensity. *: significant correlations.

Correlations between keystone species abundance and soil properties in intra-row and inter-row soils were evaluated using Spearman’s rank correlation. In intra-row soils, 91 keystone species showed significant correlations with soil properties, clustered into two groups. Cluster I (31 ASVs), dominated by Proteobacteria, was positively correlated with pH, moisture, and DOC, and was negatively correlated with NO_3_^−^-N, MBC, and MBN. Cluster II (54 ASVs), dominated by Actinobacteria, was positively correlated with TN, NH_4_^+^-N, MBC, and MBN, and was negatively correlated with pH, moisture, C:N, and DOC (Figure 9A). In inter-row soils, 81 keystone species showed significant correlations with soil properties, also clustered into two groups. Cluster I (36 ASVs), dominated by Acidobacteria, was positively correlated with TC, NO_3_^−^-N, and MBC, and was negatively correlated with pH, moisture, DOC, and MBN. Cluster II (45 ASVs), dominated by Actinobacteria, was positively correlated with C:N and negatively correlated with TN, NH₄^+^-N, and MBC (Figure 9B).

**Figure 10:**
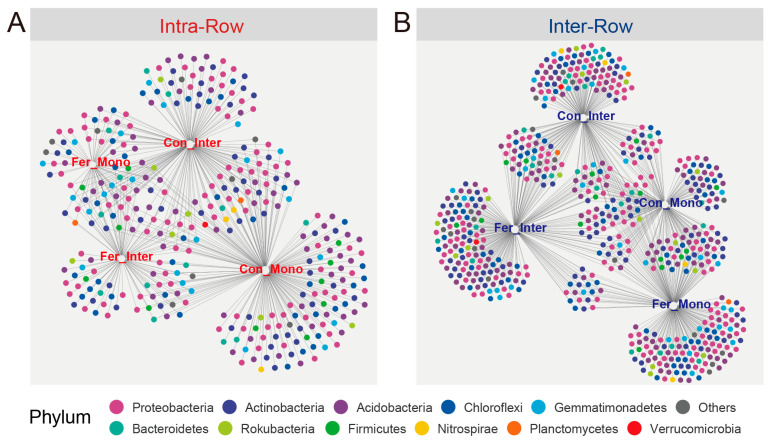
Bipartite networks display indicator species in intra-row (**A**) and inter-row (**B**) soils. Specific ASVs significantly associated with one or more treatments were selected. Circles representing individual ASVs are colored according to their phylum assignments.

By calculating the correlations between ASVs and four treatments, the indicator species in intra-row and inter-row soils were determined. These ASV abundances varied with different treatments. A total of 238 and 433 ASVs were identified as the indicator species in intra-row and inter-row soils, respectively. The results were summarized using bipartite networks (Figure 10). The indicator species associated with treatments in intra-row soils included Actinobacteria (71 ASVs, 27.95%), Acidobacteria (60 ASVs, 23.62%), Chloroflexi (36 ASVs, 14.17%), Proteobacteria (32 ASVs, 12.60%), Gemmatimonadetes (19 ASVs, 7.48%), and Bacteroidetes (12 ASVs, 4.72%; Figure 10A). In inter-row soils, indicator species were dominated by Proteobacteria (119 ASVs, 25.27%), Acidobacteria (98 ASVs, 20.81%), Actinobacteria (79 ASVs, 16.77%), Chloroflexi (57 ASVs, 12.10%), Bacteroidetes (35 ASVs, 7.43%), and Gemmatimonadetes (25 ASVs, 5.31%; Figure 10B).

We defined the ASVs as key indicator species by combining the keystone species and indicator species. A total of 42 and 41 key indicator species were obtained in intra-row and inter-row soils, respectively. Among them, 10 key indicator species were shared, including *Solirubrobacter*, *Blastococcus*, *Iamia*, *Conexibacter*, and *Lysobacter* at the genus level. In intra-row soils, 32 key indicator species were identified, including *Blastococcus*, *Lysobacter*, *Microvirga*, *Pseudarthrobacter*, *RB41*, and *Subgroup_10*. A total of 31 key indicator species were identified as *Aeromicrobium*, *Blastococcus*, *Gaiella*, *Hirschia*, *Kribbella*, *Nocardioides*, *RB41*, *Romboutsia*, *Skermanella*, *Sphingomonas*, and *SWB02* in inter-row soils (Figure S7). Moreover, correlations between key indicator species and plant height, as well as aboveground biomass, were assessed using Spearman’s rank correlation coefficient. In intra-row soils, 19 key indicator species were significantly correlated with maize height (14 negative, 5 positive), and 16 key indicator species were significantly correlated with aboveground biomass (12 negative, 4 positive). Only 4 key indicator species showed significant correlations with both maize height and aboveground biomass in inter-row soils, all of which showed a significant negative correlation with aboveground biomass (Table S4).

## 4. Discussion

Agricultural practices perturb the dynamic equilibrium of soil microbial communities to varying degrees, altering the diversity of microbial assemblages in the rhizosphere and bulk soils [34,35,76,77,78]. These perturbations not only cause structural and functional changes in soil microbial communities but also disrupt microbial interactions. Recent research has highlighted the increasing threat posed by traditional agricultural practices to soil microbiomes. These practices disrupt soil physical structure and damage the microhabitats essential for maintaining microbial diversity and functional stability [48,79,80]. Understanding the different responses of soil microbial communities to agricultural practices in rainfed agroecosystems, where abiotic challenges are increasing, is crucial for optimizing agricultural practices, preventing yield decline, and harnessing beneficial microbial functions. Identifying key microbial taxa associated with agricultural practices can provide strategic guidance for sustainable agricultural strategies that maintain soil health while increasing crop productivity.

### 4.1. Bacterial Communities Produce Significant Responses to Compartment Soils

Studies have shown significant variations in bacterial communities between the rhizosphere and bulk soils across different crops in arid farmlands [27,38,76], such as maize [40,77]. In this study, we found that the bacterial alpha diversity in the intra-row soils was significantly lower than that in the inter-row soils (Figure 5). The bacterial community structures differed significantly between the two soil compartments (Figure 6A and Figure S2A, Table S1). Meanwhile, inter-row soils exhibited higher bacterial dissimilarities compared to intra-row soils (Figure S2B), indicating greater variations in bacterial communities across samples under agricultural practices. In addition, the topological parameters of bacterial networks also differed significantly between intra-row and inter-row soils, showing disparities in co-occurrence patterns (Figure S3). Our results suggest that the differences in bacterial communities between intra-row and inter-row soils might be attributed to the “Rhizosphere Effect” and agricultural practices [81]. Plant root systems mainly shape the surrounding microenvironment by releasing specific rhizosphere exudates. This root-regulated microenvironment is stable and the nutrient conditions are simple, thereby recruiting specific soil microorganisms to colonize intra-row soils and restricting the enrichment of certain bacterial groups. This enrichment results in a decrease in species richness and an increase in the abundance of specific groups, reducing bacterial diversity. Similar results have been reported in previous studies, where plants recruit specific microorganisms from the soil to colonize the rhizosphere by releasing specific metabolites [82,83,84], limiting the enrichment of certain microorganisms through ecological niche competition and nutrients secreted [85]. In contrast, as spatial distance increases, soil bacterial communities are not only regulated by the host plants but also significantly affected by agricultural practices. The regulatory influence of host plants on bacterial communities in inter-row soils is weakened, while the direct impact of agricultural practices is enhanced, as the inter-row soil lies distant from the relatively stable microenvironment formed by plant root activities. These bacterial communities exhibit greater sensitivity to environmental changes, ultimately resulting in higher bacterial diversity and richness in inter-row soils, along with greater dissimilarity across samples compared to intra-row soils.

Moreover, soil properties in these two compartments may further explain the variations in bacterial community diversity and structure. Significant differences in soil properties were observed between intra-row and inter-row soils (Figure S1). We speculate that changes in soil properties are closely related to plant activities [86,87], which may be attributed to the absorption of nutrients and secretion of metabolites during maize growth altering the soil nutrient environment, leading to the enrichment of bacterial communities in intra-row soils [86,87]. Therefore, soil compartment was identified as the main factor affecting the variations in bacterial communities in farmland soils. Indicator species analysis showed that inter-row soils contained more species associated with agricultural practices (Figure 10). The intra-row soils harbored more indicator species significantly correlated with maize biomass, indicating that these may relate to the soil rhizosphere environment and host plants. However, very few indicator species were significantly associated with maize biomass, suggesting that inter-row soils were more affected by agricultural practices (Table S4).

### 4.2. Bacterial Communities Exhibit Significant Responses to Agricultural Practices in Different Soil Compartments

Our results indicated that agricultural practice treatments significantly affected the Shannon index and PD in inter-row soils, while no significant changes in richness, Shannon index, and PD were observed among treatments in intra-row soils (Table 2). Meanwhile, notable differences in soil properties were detected across agricultural practices, with slight changes in TC and TN in intra-row soils (Figure 2). We surmised that soil properties represent the primary drivers of alpha diversity variations [34,88]. In inter-row soils, treatments altered soil properties and disrupted microbial habitats [76,89]. Bacterial communities reacquired nutrients and ecological niches through competition, limiting the colonization of specific taxa and thus influencing alpha diversity. The results also demonstrated that agricultural practices significantly impacted soil bacterial community structure, with compartmental heterogeneity in the bacterial community variation observed between intra-row and inter-row soils. Treatments substantially altered bacterial community structures in inter-row soils, and significant differences in bacterial dissimilarities were detected among treatments (Figure 6B,C). In intra-row soils, although bacterial community structures varied significantly among treatments, no notable differences in dissimilarities existed between Con_Inter, Fer_Mono, and Fer_Inter (Figure 6B,C). Additionally, bacterial community compositions indicated that treatments altered the relative abundances of dominant phyla. Acidobacteria, Chloroflexi, Gemmatimonadetes, and Nitrospirae showed significant variations across treatments in inter-row soils (Figure 4), but only Acidobacteria and Gemmatimonadetes showed slightly significant differences in the intra-row soils. Collectively, these results revealed that treatments significantly altered bacterial community diversity and structure in inter-row soils, along with the relative abundances of dominant phyla. These treatments slightly influenced the bacterial community diversity, structure, and dominant phyla in intra-row soils. The observed differences in soil properties might contribute to the variations in bacterial communities among different treatments [27,34,35,76,78]. To further characterize treatment effects, bipartite networks were constructed between indicator species identified via indicator species analysis. In intra-row soils, 238 indicator species were identified to be associated with different agricultural practices, compared to 433 indicator species in inter-row soils (Figure 10). Inter-row soils exhibited a higher proportion of treatment-specific indicator species (Figure 10B), whereas intra-row soils showed greater overlaps with increased proportions of shared indicator species across treatments (Figure 10A). These greater overlaps of indicator species among treatments corresponded to greater similarities of bacterial communities in intra-row soils. The distribution patterns of these indicator species suggested that intra-row soils were subject to fewer agricultural practices. Therefore, the indicator species distribution collectively represented the differences in bacterial communities across treatments [90], reflecting the distinct community structures in both intra-row and inter-row soils.

### 4.3. Co-Occurrence Networks Reflect the Effects of Agricultural Practices on Soil Bacteria Communities

Co-occurrence networks were employed to investigate the interactions and ecological stability of bacterial communities within agricultural practice treatments in two soil compartments. In this study, these networks demonstrated the impacts of different treatments on bacterial co-occurrence patterns in intra-row and inter-row soils (Figure 7A,B). The topological parameters of intra-row networks under different treatments were similar (Figure 7A), showing consistent characteristics with minimal variations. Network nodes and topological features were weakly affected by treatments, with slight but significant differences in closeness centrality and eigenvector centrality among treatments. In inter-row soils, the treatment significantly affected the bacterial networks (Figure 7B). Con_Mono exhibited the highest degree, betweenness centrality, and eigenvector centrality, but the lowest closeness centrality. These results indicated that agricultural practices significantly altered bacterial co-occurrence patterns and network parameters [43,91,92,93]. Robustness analysis validated the differences in stability among networks under different treatments [43,93]. Intra-row networks remained relatively stable with slight differences, while inter-row networks showed significant stability variations. In inter-row networks, Con_Mono displayed stronger stability compared to Con_Inter, Fer_Mono, and Fer_Inter (Figure 7C). We speculate that these differences were linked to the intensity of agricultural practices. Con_Mono caused the least disruption to the soils among these four agricultural practices and had the strongest stability in bacterial communities [94,95,96]. Keystone species were identified based on node connectivity relationships. A total of 200 keystone species were identified in intra-row networks (Figure S6A) and 211 in inter-row networks (Figure S6B). In intra-row soils, 22 key indicator species were significantly correlated with maize biomass, such as *Blastococcus*, *Pseudarthrobacter*, *Iamia*, *Conexibacter*, *Microvirga*, and *Lysobacter*, whereas in inter-row soils, only 4 key indicator species showed significant correlations (Table S4). These key indicator species in the intra-row soils have also been reported in previous studies as beneficial microorganisms in farmlands [24]. For example, *Microvirga* contributes to nitrogen fixation, supplying nitrogen nutrients and enhancing plant growth, processes particularly critical in nitrogen-deficient soils [97,98]. *Lysobacter*, a plant-beneficial bacterium that shows significantly increased abundance in no-tillage or reduced-tillage soils, has a population that positively impacts yield [99,100]. *Blastococcus* enriches crop rhizospheres under diverse environmental conditions, improving soil structure to indirectly stimulate plant growth and enhance yield and drought resistance [101,102]. It is precisely due to the important roles played in various soil environments and agricultural practices that these bacteria have the potential to become effective biological indicators for evaluating agricultural productivity. The above results highlight differential responses of bacterial communities in distinct soil compartments to agricultural practices. Organic nutrients and metabolites provide carbon sources for bacterial keystone taxa in intra-row soils. The maize root environment stabilized intra-row networks, with more soil keystone taxa significantly correlated with maize biomass. In contrast, inter-row networks showed significant stability differences, where keystone taxa better reflected the effects of agricultural interventions.

Furthermore, subnetworks of keystone species with absolute correlation coefficients greater than 0.9 were constructed to reveal differences in interaction patterns among taxa. Intra-row subnetworks showed higher negative correlation ratios, possibly due to competition for nutrients and niches. In contrast, inter-row subnetworks exhibited lower negative correlation ratios. Fer_Inter exhibited the highest negative correlation ratios, while Con_Mono had the lowest (Figure 8). These subnetworks indicated that inter-row keystone species rely on positive interactions for resource acquisition, which are also influenced by agricultural practices [103]. Agricultural practices disrupt the original soil structure, nutrient and water supply, and microenvironment homogeneity in inter-row soils. This leads to the enrichment of different microbial groups, each occupying specific ecological niches for survival, reducing competition and enhancing cooperation through functional division of labor. These results underscored compartment-specific responses of bacterial taxa to agricultural strategies, highlighting the roles of root–microbiome interactions in stabilizing intra-row bacterial communities and the influence of agricultural practice treatments in shaping inter-row differential networks [104,105]. Microbial interactions are driven by resources, metabolism, environmental factors, and interspecific relationships. Positive correlations primarily involve cooperative symbiosis, while negative correlations primarily involve competitive antagonism, collectively maintaining the dynamic equilibrium of ecosystems.

## 5. Conclusions

Soil microbial communities serve as core functional units of modern precision agriculture systems, and their spatial heterogeneity is crucial for constructing soil evaluation frameworks. Accurate frameworks provide a foundation for optimizing tillage patterns in agricultural practices. Our findings clarify how soil microbial communities spatially respond to agricultural practices and identify key indicator taxa significantly correlated with maize biomass across soil compartments. These key indicator species have also been discovered in diverse environments, but their suitability to various crop systems and soil types still needs more experiments for verification and further exploration. By developing a spatial–microbial evaluation model, the comprehensive assessment capacity of agricultural practices can be enhanced. This research advances understanding of microbial roles in soil evaluation and offers guidance for improving soil health and sustainable agricultural development.

## Figures and Tables

**Table 1:** Variations in maize height and aboveground biomass across treatments. Significance was assessed using the Kruskal–Wallis test, and different letters in the same column denote significant differences among treatments.

	Maize Height (cm)	Aboveground Biomass (g Plant^−1^)
Con_Mono	136.15 ± 4.78 a	99.50 ± 4.01 a
Con_Inter	185.25 ± 4.07 b	150.35 ± 8.26 b
Fer_Mono	211.05 ± 5.95 c	146.80 ± 7.52 b
Fer_Inter	235.95 ± 4.68 d	208.95 ± 15.78 c

All values are shown as mean ± standard error.

**Table 2:** Alpha diversity of bacterial communities in different treatments. Significance of differences among treatments in intra-row and inter-row soils was assessed separately using the Kruskal–Wallis test. Different letters in the same row indicate significant differences among treatments.

Alpha Diversity		Con_Mono	Con_Inter	Fer_Mono	Fer_Inter
Richness	Intra-Row	860.50 ± 46.04 a	904.75 ± 70.81 a	748.00 ± 53.99 a	818.25 ± 73.49 a
	Inter-Row	1262.13 ± 147.10 a	1436.13 ± 160.21 a	988.88 ± 84.89 a	1407.63 ± 129.98 a
Shannon	Intra-Row	6.27 ± 0.04 a	6.32 ± 0.05 a	6.16 ± 0.09 a	6.19 ± 0.11 a
	Inter-Row	6.59 ± 0.19 ab	6.60 ± 0.13 ab	6.31 ± 0.08 a	6.78 ± 0.09 b
PD	Intra-Row	89.86 ± 3.16 a	96.96 ± 3.43 a	88.26 ± 4.09 a	93.17 ± 4.98 a
	Inter-Row	126.31 ± 7.70 ab	136.14 ± 8.13 b	99.83 ± 5.43 a	127.96 ± 7.70 ab

All values are shown as mean ± standard error.

## Data Availability

The original contributions presented in this study are included in the article. Further inquiries can be directed to the corresponding authors.

## Supplementary Materials

The following supporting information can be downloaded at: https://www.mdpi.com/article/10.3390/microorganisms13051160/s1. Figure S1: Soil properties between intra-row and inter-row soils; Figure S2: General patterns of bacterial communities between compartment soils; Figure S3: Co-occurrence patterns of bacterial communities in intra-row and inter-row soils; Figure S4: Taxonomic composition of major modules in intra-row and inter-row networks; Figure S5: Topological features of co-occurrence networks across treatments in intra-row and inter-row soils; Figure S6: Node connectivity attributes in intra-row and inter-row networks; Figure S7: Distributions of keystone and indicator species; Table S1: Effects of compartment and treatment on Bray–Curtis distance of soil bacterial communities using PERMANOVA and ANOSIM; Table S2: Effects of treatment on Bray–Curtis distance of soil bacterial communities in intra-row and inter-row samples using PERMANOVA and ANOSIM; Table S3: Topological parameters of co-occurrence networks between intra-row and inter-row soils; Table S4: Key indicator species significantly associated with plant height and aboveground biomass in intra-row and inter-row soils.
