# Supplementary material for: Spatial Heterogeneity in Soil Microbial Communities Impacts Their Suitability as Bioindicators for Evaluating Productivity in Agricultural Practices
Source: Microorganisms. 2025 May 20;13(5):1160. doi: 10.3390/microorganisms13051160 (PMC12113728; doi:10.3390/microorganisms13051160)
Supplement: Supplementary file 1 [file microorganisms-13-01160-s001.zip › microorganisms-3599759-supplementary.pdf]

Supplementary material

Spatial Heterogeneity in Soil Microbial Communities Impacts Their Suitability as Bioindicators for Evaluating Productivity in Agricultural Practices

Guoqiang Li, Xuanjing Li, Ting Jin, Muyilan Jiang, Peng Shi * and Gehong Wei *

State Key Laboratory for Crop Stress Resistance and High-Efficiency Production, Shaanxi Key Laboratory of Agricultural and Environmental Microbiology, College of Life Sciences, Northwest A&F University, Yangling 712100, China; guoqiangli@nwafu.edu.cn (G.L.); 15837122712@163.com (X.L.); m15284435216@163.com (T.J.); 2019013669jmyl@nwafu.edu.cn (M.J.)

* Correspondence: shipeng27@nwafu.edu.cn (P.S.); weigehong@nwsuaf.edu.cn (G.W.); Tel.: +86-135-7227-7787 (P.S.)

Table S1. Effects of compartment and treatment on Bray-Curtis distance of soil bacterial communities using PERMANOVA and ANOSIM. Significant effects are indicated in bold with $p < 0.05$.

Bray-Curtis distance	PERMANOVA		ANOSIM	
	R^2	p	R	p
Compartment	0.052	**< 0.001**	0.146	**< 0.001**
Treatment	0.081	**< 0.001**	0.095	**< 0.001**

Table S2. Effects of treatment on Bray-Curtis distance of soil bacterial communities in intra-row and inter-row samples using PERMANOVA and ANOSIM. Significant effects are indicated in bold with $p < 0.05$.

Bray-Curtis distance	PERMANOVA		ANOSIM	
	R^2	p	R	p
Intra-Row	0.143	**< 0.001**	0.203	**< 0.001**
Inter-Row	0.183	**< 0.001**	0.221	**< 0.001**

Table S3. Topological parameters of co-occurrence networks between intra-row and inter-row soils. Significance of these difference was assessed separately using Kolmogorov-Smirnov tests under different treatments. Asterisks denote significant differences. ***: $p < 0.001$; **: $p < 0.01$. Non-significant difference is not marked with asterisk.

Topological features	Intra-Row *vs.* Inter-Row			
	Degree	Betweenness	Closeness	Eigenvector
Con_Mono	0.400***	0.383***	0.809***	0.563***
Con_Inter	0.260***	0.453***	0.730***	0.734***
Fer_Mono	0.144**	0.275***	0.638***	0.401***
Fer_Inter	0.027	0.130**	0.298***	0.540***

Table S4. Key indicator species significantly associated with plant height and aboveground biomass in intra-row and inter-row soils. Correlations between key indicator species and plant height, as well as aboveground biomass, were assessed using Spearman's rank correlation coefficient. Values > 0 represent positive correlations, while values < 0 represent negative correlations. Asterisks denote significant positive or negative correlations. ***: $p < 0.001$; **: $p < 0.01$; *: $p < 0.05$. "--": not identified.

	ASV ID	Phylum	Class	Order	Family	Genus	Maize Height	Aboveground Biomass
Intra-Row	ASV_1600	Acidobacteria	Blastocatellia	Pyrinomonadales	Pyrinomonadaceae	*RB41*	-0.49**	-0.45*
	ASV_335	Acidobacteria	Blastocatellia	Pyrinomonadales	Pyrinomonadaceae	*RB41*	-0.55**	-0.56***
	ASV_92	Acidobacteria	Holophagae	Subgroup_7	--	--	0.35	0.36*
	ASV_305	Acidobacteria	Subgroup 6	--	--	--	0.40*	0.27
	ASV_566	Acidobacteria	Thermoanaerobaculia	Thermoanaerobaculales	Thermoanaerobaculaceae	*Subgroup_10*	-0.43*	-0.26
	ASV_451	Actinobacteria	Acidimicrobiia	Microtrichales	Iamiaceae	*Iamia*	-0.42*	-0.43*
	ASV_202	Actinobacteria	Acidimicrobiia	Microtrichales			-0.28	-0.39*
	ASV_30	Actinobacteria	Actinobacteria	Frankiales	Geodermatophilaceae	*Blastococcus*	-0.49**	-0.49***
	ASV_46	Actinobacteria	Actinobacteria	Frankiales	Geodermatophilaceae	*Blastococcus*	-0.42*	-0.43*
	ASV_7	Actinobacteria	Actinobacteria	Frankiales	Geodermatophilaceae	*Blastococcus*	-0.48**	-0.38*
	ASV_13	Actinobacteria	Actinobacteria	Micrococcales	Micrococcaceae	*Pseudarthrobacter*	-0.25	-0.40*
	ASV_436	Actinobacteria	Actinobacteria	Micrococcales	Micrococcaceae	--	-0.39*	-0.25
	ASV_587	Actinobacteria	Thermoleophilia	Solirubrobacterales	Solirubrobacteraceae	*Conexibacter*	-0.38*	-0.33
	ASV_289	Chloroflexi	Gitt-GS-136	--	--	--	-0.39*	-0.47**
	ASV_44	Chloroflexi	JG30-KF-CM66	--	--	--	-0.42*	-0.40*
	ASV_541	Entotheonellaeota	Entotheonellia	Entotheonellales	Entotheonellaceae	--	0.39*	0.35*
	ASV_108	Gemmatimonadetes	Gemmatimonadetes	Gemmatimonadales	Gemmatimonadaceae	--	0.43*	0.47**
	ASV_45	Gemmatimonadetes	Gemmatimonadetes	Gemmatimonadales	Gemmatimonadaceae	--	0.45**	0.31
	ASV_68	Gemmatimonadetes	Gemmatimonadetes	Gemmatimonadales	Gemmatimonadaceae	--	-0.43*	-0.45*
	ASV_199	Proteobacteria	Alphaproteobacteria	Rhizobiales	Beijerinckiaceae	*Microvirga*	0.40*	0.43*
	ASV_448	Proteobacteria	Gammaproteobacteria	Xanthomonadales	Xanthomonadaceae	*Lysobacter*	-0.42*	-0.36*
	ASV_904	Proteobacteria	Gammaproteobacteria	Xanthomonadales	Xanthomonadaceae	*Lysobacter*	-0.48**	-0.32
Inter-Row	ASV_99	Acidobacteria	Subgroup_6	--	--	--	-0.23	-0.36*
	ASV_326	Actinobacteria	Acidimicrobiia	IMCC26256	--	--	-0.35*	-0.43*
	ASV_21	Actinobacteria	MB-A2-108	--	--	--	-0.23	-0.36*
	ASV_130	Bacteroidetes	Bacteroidia	Cytophagales	Microscillaceae	--	-0.26	-0.36*

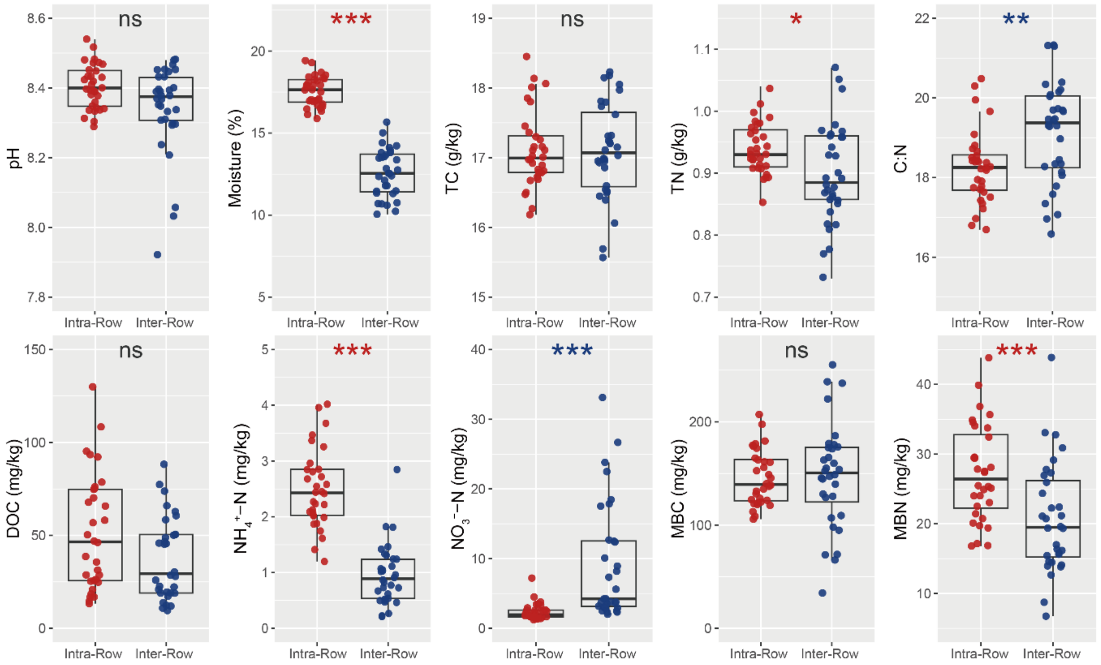

Figure S1. Soil properties between intra-row and inter-row soils. Significance of these differences was assessed using the Wilcoxon rank sum test. ***: $p < 0.001$; *: $p < 0.05$; ns: non-significant. TC: total carbon; TN: total nitrogen; C:N: total carbon/nitrogen ratio; DOC: dissolved organic carbon; NH_4^+-N: ammonium nitrogen; NO_3^--N: nitrate nitrogen; MBC: microbial biomass carbon; MBN: microbial biomass nitrogen.

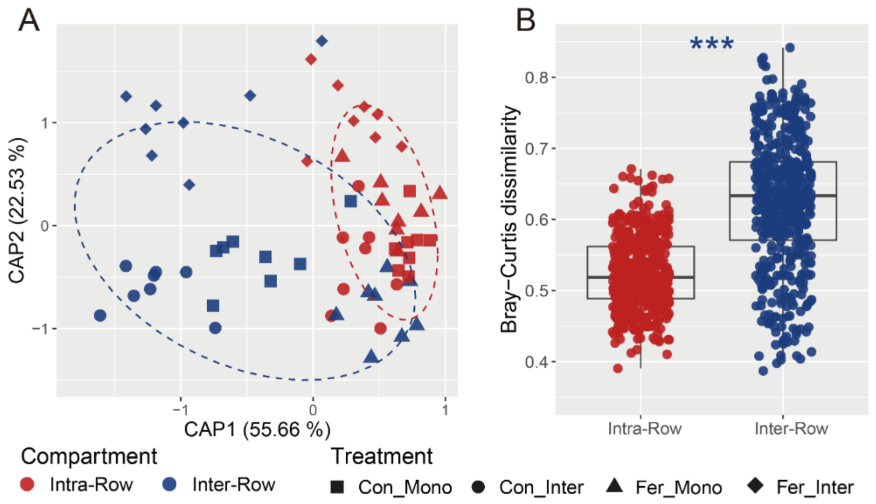

Figure S2. General patterns of bacterial communities between compartment soils. (A) CAP shows the treatment patterns in intra-row and inter-row soils. The CAP analyses were constrained by “compartment” and "treatment". Colored circles represent different compartment soils, while shapes represent the treatments. 80% confidence ellipses are shown around each compartment. (B) Bray-Curtis dissimilarity of bacterial communities between intra-row and inter-row soils. Blue asterisks indicate a significantly higher dissimilarity in inter-row soils (Wilcoxon rank sum test). ***: $p < 0.001$.

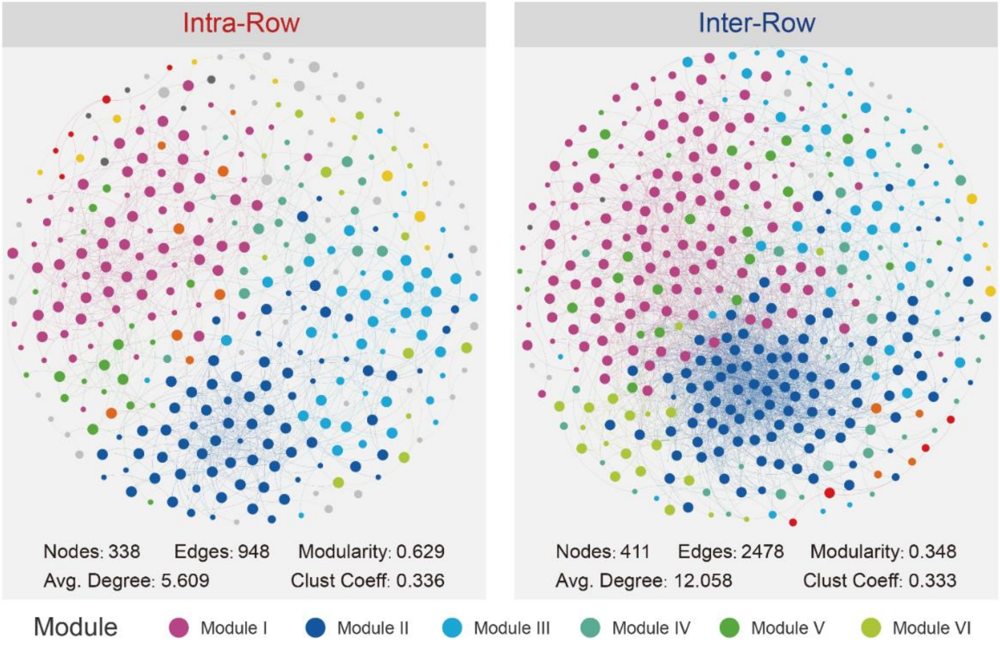

Figure S3. Co-occurrence patterns of bacterial communities in intra-row and inter-row soils. Networks were constructed by calculating correlations among ASVs ($|\rho| > 0.6$, $p < 0.001$). Nodes and edges are colored according to clusters (modules). Node sizes represent the degree of connections. Topological parameters (nodes, edges, modularity, average degree, and clustering coefficient) are presented at the bottom of each plot.

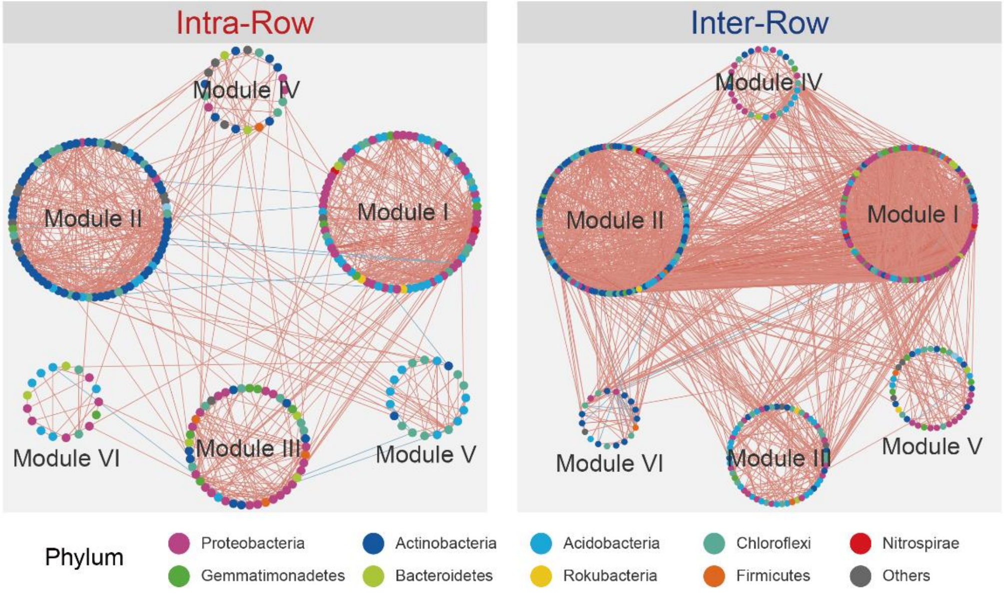

Figure S4. Taxonomic composition of major modules in intra-row and inter-row networks. Six modules with the highest node counts are shown. Nodes are colored according to taxonomic classification at the phylum level. Colored edges represent positive (red) and negative (blue) correlations.

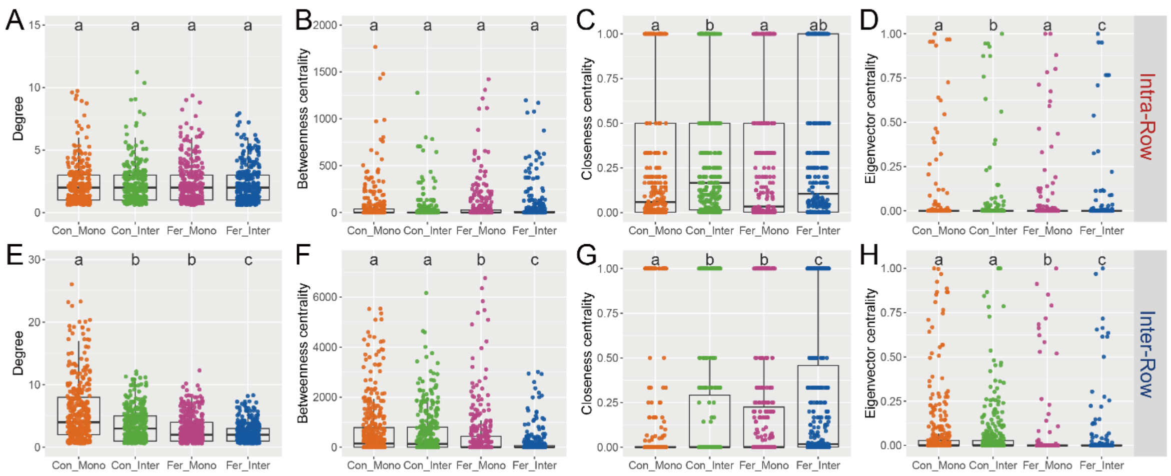

Figure S5. Topological features of co-occurrence networks across treatments in intra-row (A-D) and inter-row soils (E-H). Significance of these differences was assessed using the Kruskal-Wallis test. Different letters indicate significant differences between treatments.

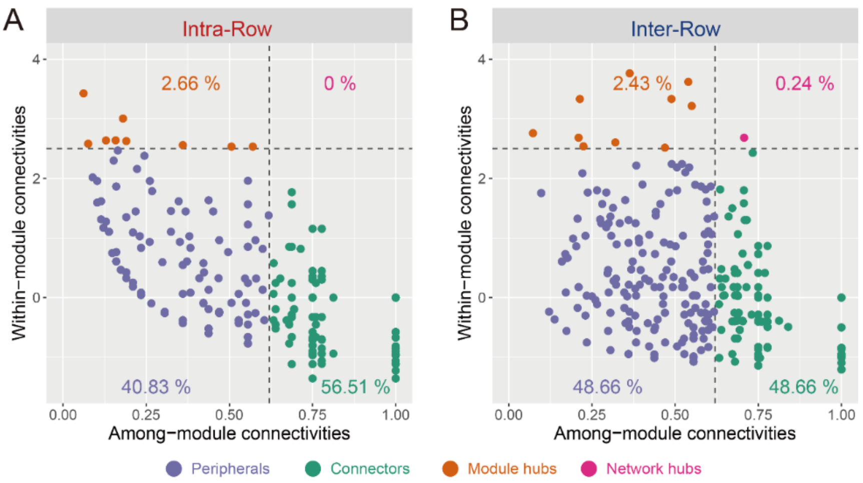

Figure S6. Node connectivity attributes in intra-row (A) and inter-row (B) networks. Circles are colored according to node attributes. Nodes classified as connectors, module hubs, and network hubs were defined as keystones.

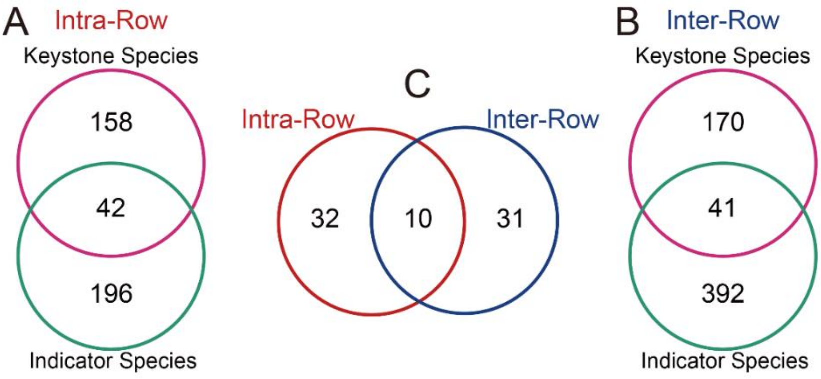

Figure S7. Distributions of keystone and indicator species. (A-B) Shared and unique ASVs of keystone and indicator species in intra-row (A) and inter-row (B) soils. (C) Distributions of key indicator species in intra-row and inter-row soils.
